# Thrombin generation assay and transmission electron microscopy: a useful combination to study tissue factor-bearing microvesicles

**DOI:** 10.3402/jev.v2i0.19728

**Published:** 2013-03-18

**Authors:** Damien Gheldof, Julie Hardij, Francesca Cecchet, Bernard Chatelain, Jean-Michel Dogné, François Mullier

**Affiliations:** 1 Department of Pharmacy, Namur Research Institute for LIfe Sciences, Namur Thrombosis and Hemostasis Center (NTHC), University of Namur, Belgium; 2 Haematology Laboratory, Namur Research Institute for LIfe Sciences, Namur Thrombosis and Hemostasis Center (NTHC), CHU Mont-Godinne, Université Catholique de Louvain, Belgium; 3 Research Centre in Physics of Matter and Radiation, Namur Research Institute for LIfe Sciences, University of Namur, Belgium

**Keywords:** tissue factor, microvesicles, cancer, thrombin generation

## Abstract

**Introduction:**

Patients with cancer have a 7- to 10-fold increased risk of developing venous thromboembolism. Circulating microvesicles could be a useful predictive biomarker for venous thromboembolism in cancer. Validated and standardised techniques that could be used to determine the complete microvesicle phenotype are required.

**Objectives:**

These were two-fold: a) to characterise tissue factor (TF)-bearing microvesicles released by cultured breast cancer cells MDA-MB-231 by flow cytometry (FCM), transmission electron microscopy (TEM) and thrombin generation assay (TGA); and b) to validate the sensitivity and variability intra/inter-assay of TGA as a useful method to study the procoagulant activity (PCA) of microvesicles.

**Methods:**

Cultured breast cancer cells MDA-MB-231 were incubated for 45 minutes at 37°C. Samples were then centrifuged or not at 4,500 g for 15 minutes, and cells and MVs or MV-containing supernatants were used for TEM, FCM and TGA. In activity assays, microvesicles (i.e. cell-depleted supernatants) were incubated with anti-TF antibodies or with annexin V to assess the contribution of TF and phospholipids to the PCA. Alternatively, supernatants were filtered through 0.1, 0.22, 0.45 or 0.65 µm membranes and subjected to TGA.

**Results:**

The majority of the PCA was associated with microvesicles smaller than 0.1 µm, and the mean microvesicle size estimated by TEM after 10,000 g centrifugation was 121±54 nm with a majority of vesicles between 100 and 200 nm. Microvesicles derived from 5,000 MDA-MB-231cells/ml were sufficient to significantly increase the thrombin generation of normal pooled plasma.

**Conclusions:**

TEM, FCM and filtration coupled to TGA represent a useful combination to study the PCA of TF-bearing microvesicles, whatever their size. And it will be interesting to implement these techniques in patients.

Venous thromboembolism (VTE) is responsible for 15% of deaths in cancer patients (1–3). Therefore, thromboprophylaxis for hospitalised cancer patients is recommended (4). However, individual risk factors cannot identify a group of outpatients at highest risk for VTE that would benefit from thromboprophylaxis (4). Several studies strongly suggest that microvesicles (MVs) harbouring tissue factor (TF-MVs) activity may have prognostic value in identifying cancer patients with increased risk of VTE (5). MVs are small membrane vesicles shed by most normal and/or tumour cells constitutively or following activation or apoptosis (6, 7). Based on the size and mechanism of synthesis, MVs are currently divided into exosomes and microparticles (MPs) (8). The diameter of exosomes is comprised between 30 and 100 nm, whereas MPs have a size comprised between 100 nm and 1 µm. However, this definition is not generally accepted due to technological limitations in size measurement (8). MVs may present TF and negatively charged phospholipids (PL) such as the phosphatidylserine on their membrane. These elements are thought to be implicated in the PCA (9). In several types of cancers, including breast and pancreatic cancers, TF expression is associated with a prothrombotic phenotype and correlates with grade and tumour progression (6, 7, 10). The TF is present in plasma under 2 major forms: a full-length TF that may be anchored at MVs surface (TF-MVs) or an alternative spliced soluble protein. TF associated with MVs is considered as the main form exhibiting PCA (2, 11, 12). MVs are defined by size, concentration, morphology, biochemical composition, cellular origin and activity. Numerous techniques have been described to detect and/or characterise the MVs (8). However, no technique is able to provide all MV characteristics. Consequently, the combination of different techniques is required for a complete description of MVs (9). For example, flow cytometry (FCM), which is the most used technique to study MVs, suffers from a lack of sensitivity to small-sized MVs and gives no information about the TF and/or phospholipid-mediated PCA. Thrombin generation assay (TGA) can be used to specifically assess the impact of TF-MV on coagulation. However, this technique is limited by absence of standardisation and by the unknown sensitivity to low levels of TF due to presence of TFPI in plasma (13, 14).

It is therefore essential to validate and standardise a panel of techniques that could be used to determine the complete MVs phenotype and to study the effects of pre-analytical steps on MVs’ conformation (potential fragmentation or aggregation).

The objectives of this study were therefore to (a) to characterise tumour-cell-derived MVs released by cultured MDA-MB-231 breast cancer cells with FCM, transmission electron microscopy (TEM) and TGA; and (b) to validate the intra/inter-assay sensitivity and variability of TGA as a useful method to analyse MVs PCA.

## Material and methods

### Cell culture

Adherent MDA-MB-231 breast cancer cells (ATCC number HTB-26) were obtained from the American Type Culture Collection (Manassas, VA, USA) and were cultured in RPMI-1640 medium (Lonza, Verviers, Belgium) supplemented with 10% foetal bovine serum (FBS) (Lonza, Verviers, Belgium). Cells were maintained in a 5% CO_2_ humidified atmosphere at 37°C. Natrium bicarbonate was set at 1.5 g/L to control the pH level. Cell viability was controlled by the trypan blue exclusion test.

### Preparation of normal pooled plasma and platelet-free plasma

Normal pooled plasma (NPP) was prepared as previously described (15). Platelet-free plasma was obtained by a first centrifugation once at 2,500 g for 15 minutes at room temperature (Heraeus Multifuge 1S-R, Sysmex Benelux, Etten-Leur, The Netherlands) with a light break only, within one hour after sampling. The platelet-poor plasma was collected and transferred into a polypropylene haemolysis tube with a micropipette. Aspiration was stopped 1 cm above the buffy-coat while avoiding the buffy-coat. Platelet-poor plasma was centrifuged a second time at 2,500 g for 15 minutes at room temperature. The platelet-free plasma was collected into a fresh tube using a micropipette, while leaving about 100 µl at the bottom of the tube.

### In vitro generation of MVs

**Fig. 1:**
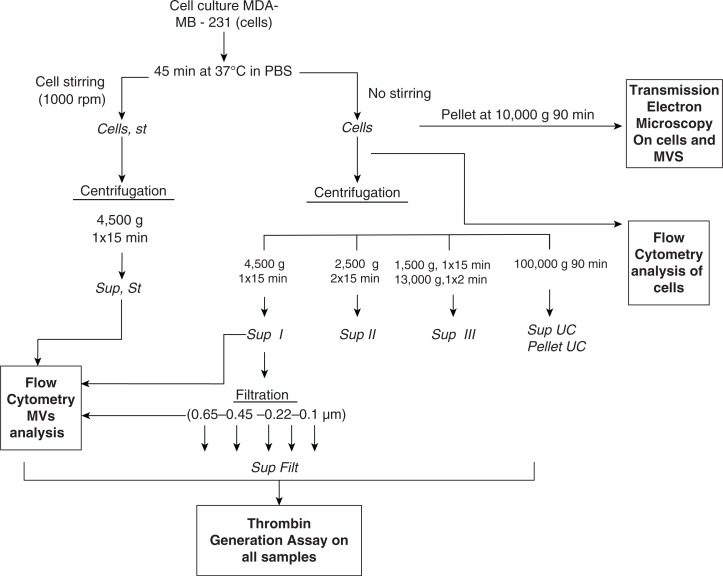
Sample preparation. All supernatants were freshly used for TEM, FCM and TGA. PBS: Phosphate Buffer Saline. Sup: Supernatant. Filt: Filtrate. UC: ultracentrifugation.

A modified version of the protocol reported by Davila et al. was performed (11). Cells were adjusted to the desired concentrations (600,000 cells/ml for TGA and TEM) in PBS and then incubated for 45 minutes at 37°C without any stirring. Samples were then centrifuged or not at 4,500 g for 15 minutes (Fig. 1). Afterwards, cells and MVs or fresh MV-containing supernatants devoid of cells were used for TEM, FCM and TGA. PBS was used as a negative control.

### Preparation of samples for TEM observation: tumour MVs visualisation and sizing by TEM

**Fig. 2:**
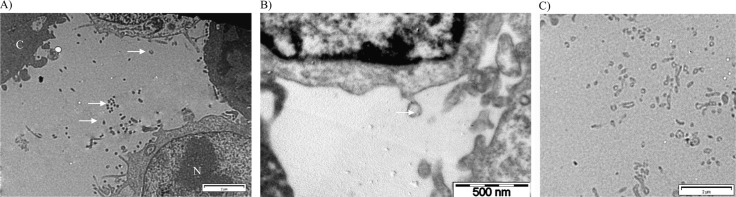
Transmission electron microscopy pictures of cells with derived MVs (white arrows) after ultracentrifugation at 10,000 g during 90 minutes. A) Scale bar=2 µm. N: nucleus C: cytoplasm; B) scale bar=500 nm; C) scale bar=2 µm.

One millilitre of PBS containing 600,000 MDA-MB-231 cells and their secreted MVs was centrifuged at 10,000 g for 90 minutes at 4°C to form a pellet (Fig. 1). The pellet was fixed for TEM observation during 2.5 hours in 300 µl of 2.5% glutaraldehyde in 0.1 M cacodylate buffer. Samples were then washed using 0.2 M cacodylate buffer. Osmium tetraoxide was added during 1 hour as contrasting agent. Samples were further washed with 0.2 M cacodylate buffer and dehydrated with successive baths of alcohol from 30° to 100°. Impregnation of samples in resin LX 112 was then done. After resin polymerization, samples were cut with an ultra-microtome (LKB, Bromma 8800^®^), mounted on a grid and stained with heavy metal salts (uranyl acetate and lead citrate). The samples were observed using Tecnai 10 TEM (FEI, Eindhoven, The Netherlands; resolution of about 5 nm at 80 kV). The MV size determination was performed semi-automatically on high magnification TEM pictures (Fig. 2b) with the Image J software (Maryland, USA). One hundred MVs were sized (16). The result was expressed as mean±SD with range in parentheses.

### Counting and expression of TF and MUC-1 by FCM

Quantification of MVs and expressions of TF and Mucin-1 (MUC-1) on MDA-MB-231 cells and MVs were carried out by FCM as mentioned in supplementary information. MUC-1 was chosen since it has been shown that TF-MV activity of patients with breast cancer who presented with acute VTE, correlated with the presence in the blood of MV expressing the epithelial antigen MUC1 (17).

### Measurement of the PCA of MVs by thrombin generation assay

PCA was measured by TGA according to a modified version of the method developed by Hemker (18). Total cell suspensions or cell-depleted in vitro-generated MV fractions from 600,000 cells/ml were used as the source of TF and phospholipids. Eighty microlitre of NPP and 20 µl of cell suspensions or MV fractions were mixed in a 96-well microtitre plate (Thermo Immulon 2HB, USA) and were incubated for 10 minutes at 37°C. PBS was used as a negative control. Early experiments are tested with or without addition of 20 µl of MP reagent, which correspond to 4 µM of phospholipids (Thrombinoscope BV). The detailed protocol has been described elsewhere (15).

All the experiments were performed in triplicate. The results were expressed as means±SD. All parameters were calculated using software thrombinoscope. The percentage of PCA was calculated on the lagtime reduction by MVs.

Variability of TGA was measured according to the previously described method using 80 µL of NPP+20 µL PBS. The assays were conducted in triplicate on 6 independent runs with the same operator. Mean intra-assay variation and inter-assay variability for lagtime and peak were determined. Mean intra-assay variation corresponds to the mean coefficient of variation for the triplicates (n=6). Inter-assay variation corresponds to the coefficient of variation for the mean value of triplicates between the 6 independent runs.

### Effect of MVs size on PCA

MV fractions were filtered through 0.1, 0.22, 0.45 or 0.65 µm membranes (Ultrafree-MC; Amicon, Bedford, MA, USA) following the manufacturer's instructions. Supernatant (Sup) and filtrates (Filt) were compared by TGA and FCM (1 experiment in triplicate).

### Contribution of TF and phospholipids to MVs PCA

For contribution of TF, MVs derived from 600,000 cells/ml were pre-incubated with various monoclonal antibodies (mAbs) (10 µg/ml, optimal final concentration) for 20 minutes at room temperature before TGA was performed.

The clone HTF-1 (BD Biosciences, Erembodegem, Belgium) was used as TF activity neutralizing Ab (19). The clone TF9-10H10 (Merck, Darmstadt, Germany) and IgG1 (BD Biosciences, Erembodegem, Belgium) were tested as a non-function-blocking antibody (20) and isotypic control antibody, respectively.

The phospholipid-mediated activity was blocked with annexin V (Sigma-Aldrich, Bornem, Belgium) (21). Samples were pre-incubated with annexin V (0.5 µM, final concentration) for 20 minutes at room temperature before TGA was performed (11). Three independent experiments were performed in triplicate.

### Effect of ultracentrifugation on PCA

MVs derived from 100,000 cells/ml were separated from the cells by centrifugation at 4,500 g during 15 minutes. The collected supernatants, which contain only MVs, were ultracentrifuged at 100,000 g during 90 minutes to pellet the MVs. MVs before ultracentrifugation (Sup), pellet of MVs (Pellet UC) and supernatant after ultracentrifugation (Sup UC) were tested in TGA.

### Effect of stirring and centrifugation on MV PCA

The standard protocol described above for the in vitro generation of MVs is described in Fig. 1. In preliminary experiments, our standard protocol without stirring was compared to that proposed by Davila (11), which includes stirring (at 1,000 rpm) on an aggregometer during the 45 minutes of cell incubation in PBS. The cell supernatants were used for TGA.

Three centrifugation protocols to eliminate cells were also compared using TGA at 600,000 cells/ml: (a) simple centrifugation at 4,500 g for 15 minutes (11); (b) double centrifugation at 2,500 g for 15 minutes; and (c) double centrifugation at 1,500 g for 15 minutes and at 13,000 g for 2 minutes. Three independent experiments were performed in triplicate.

### Effect of cell concentration on MVs PCA

To determine the sensitivity of TGA, the impact of cell concentration on TGA was assessed at 100; 500; 1,000; 2,500; 5,000; 10,000; and 20,000 cells/ml. Three independent experiments were performed in triplicate.

### Statistics

Comparison between different conditions was performed using the Kruskal–Wallis test on MedCalc software (version 12.2.1.0). If the Kruskal–Wallis test is positive (p<0.05), then MedCalc performs a test for pairwise comparison of subgroups according to Conover (22).

## Results

### Characterisation of MVs derived from MDA-MB-231 cells

**Fig. 3:**
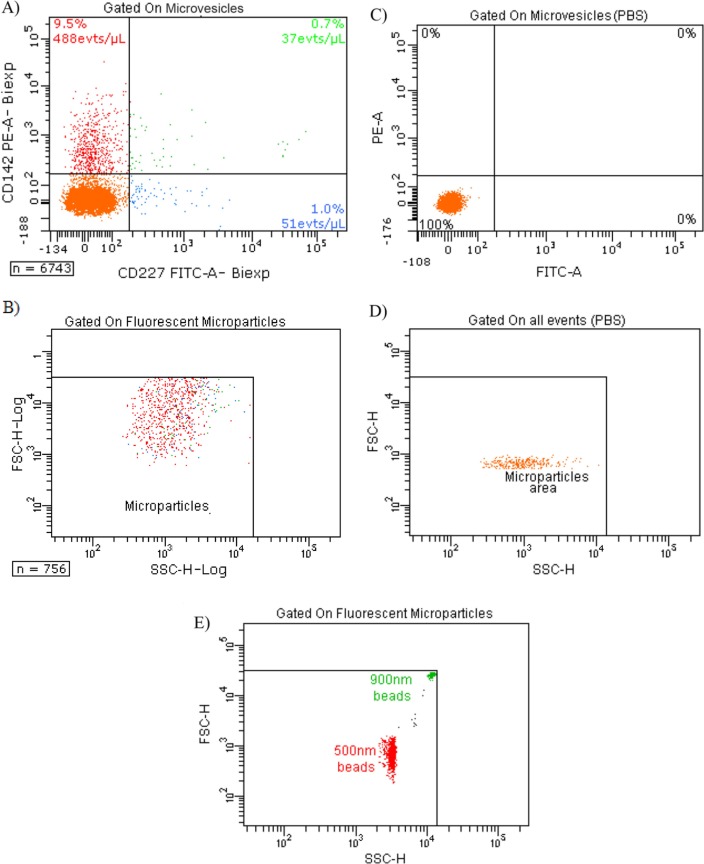
Expression of TF (CD142) and MUC-1 (CD227) on MVs depleted of cells. A) Tumour microparticle analysis. Dual fluorescence analysis of MDA-MB-231 MVs stained with CD227 fluorescein isothiocyanate (FITC) (FL1) and CD142– phycoerythrin (PE) (FL2). CD142+ CD227+ MVs are represented as green dots, CD142+ CD227– MVs as red dots, CD227+ CD142− MVs as blue dots and background noise or other MVs as orange dots. Percentage and absolute number (/µl) of each subpopulation are indicated; B) backgating of CD142+ CD227+ MVs (green dots), CD142+ CD227– MVs (red dots) and CD227+ CD142– MVs (blue dots) on FSC log-SSC log cytogram; C) expression of TF (CD142) and MUC-1 (CD227) on PBS without MVs; D) FSC-SSC of control PBS without MVs labelled similarly with CD142 and CD227. E) FSC-SSC dot plot of 500 and 900 nm beads used to gate on MVs.

The different experimental settings used in this work are schematized in Fig. 1. Images of MDA-MB-231 cells with TEM after 10,000 g centrifugation showed that these tumour cells spontaneously shed small MVs from their membrane (Fig. 2). The mean size of MVs after centrifugation of the cells and MVs was 121±54 nm (range: 57 nm to 440 nm). The size distribution was not statistically different when we ultracentrifuged cells with MVs at 100,000 g. However, we cannot provide a picture of MVs after ultracentrifugation because of the insufficient amount of the pellets. Concerning their membrane protein surface expression, MDA-MB-231 cells were strongly positive for both MUC-1 (=CD227) and TF (=CD142) (Fig. S1). Ten percent of all MVs were positive for TF expression, and only 1.7% were positive for MUC1 (Fig. 3). The final MVs concentration in MDA samples (cells and MVs) was 1,199 MVs/µl (Table I).

**Table I:** Flow cytometry concentration of MVs MUC-1+/TF−, MVs MUC-1-/TF+ and MVs MUC-1+/TF+ (/µl) in: cells and MPs (Cells), MDA-MB-231 supernatant (Sup), MDA-MB-231 supernatant produced with stirring (Sup, St), Sup filtered through 0.65 and 0.45 µm membranes (Sup Filt 0.65 µm and Sup Filt 0.45 µm) and PBS as control

Sample	MVs MUC-1+/TF− (/µl)	MVs MUC-1−/TF+ (/µl)	MVs MUC-1+/TF+ (/µl)
Cells	86	1014	99
Sup	44	60	30
Sup, St	39	128	15
Sup Filt 0.65 µm	47	25	15
Sup Filt 0.45 µm	48	21	17
PBS	0	0	0

### Procoagulant activity of MDA-MB-231 cells-derived MVs

**Fig. 4:**
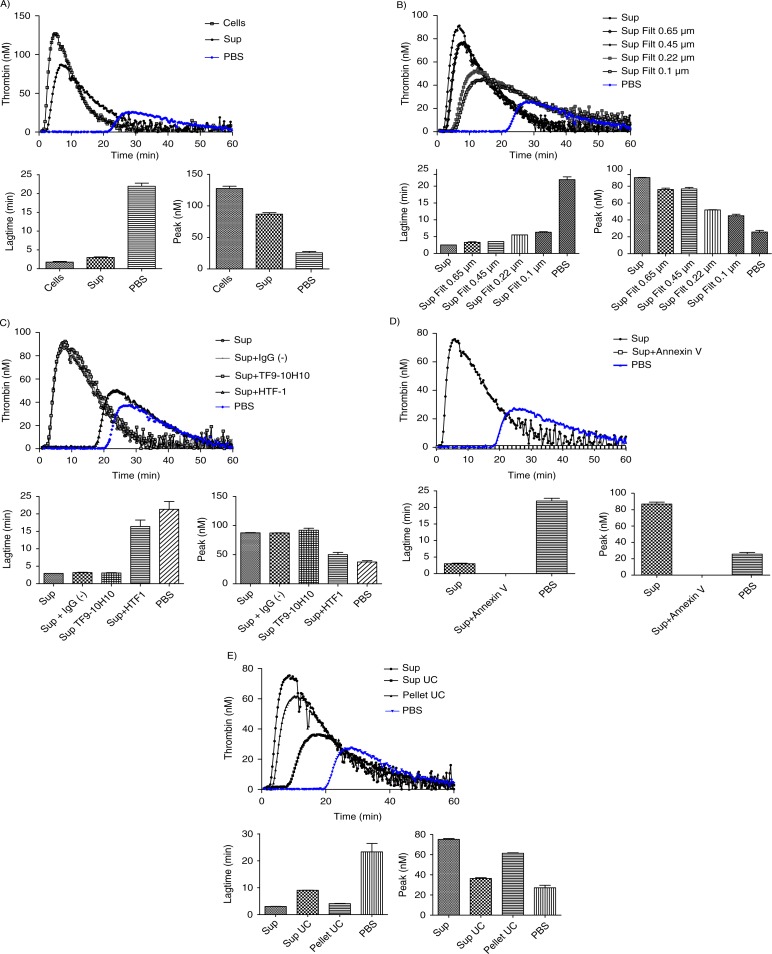
Curves of thrombin generation experiments and histograms of lagtime and peak parameters. In control condition, NPP is spiked with PBS. Curve data are presented as means of n=3. All the results are representative of 3 independent experiments. A) NPP spiked with MDA-MB-231 cells (Cells) or supernatant from MDA-MB-231 cells (Sup). The supernatant shows only slightly reduced PCA as compared to cell+supernatant; B) NPP spiked with supernatant from MDA-MB-231 cells (Sup), filtered or not through membranes with various sizes (Sup Filt 0.1 µm/0.22 µm/0.45 µm/0.65 µm). Filtration through 0.65 or 0.45 µm reduces only slightly the PCA, whereas filtration through 0.22 and 0.1 µm leads to stronger reduction in PCA; C) procoagulant effect of MVs (Sup) pre-incubated with or without HTF-1 (TF-blocking Ab), TF9-10H10 (TF-non-blocking Ab) or isotypic control antibodies at 10 µg/ml. HTF-1 strongly inhibits PCA of MV-containing supernatant; D) procoagulant effect of MVs (Sup) pre-incubated with or without annexin V at 0.5 µM. Annexin V abolishes the PCA activity of the MV-containing supernatant; E) procoagulant effect of MVs derived from 100,000 cells (Sup), of the pellet obtained after ultracentrifugation of MVs (pellet UC Sup) and of the supernatant obtained after ultracentrifugation of MVs (Sup UC).

MDA-MB-231 cells and their derived MVs (“MDA” sample) significantly boosted up the generation of active thrombin when compared to the PBS control curve (Fig. 4a), as shown by the 10-fold reduction of lagtime. MDA-MB-231 supernatant (“Sup MDA” sample), which only contains MVs, also enhanced thrombin activity compared to the control as indicated by the 9-fold lagtime reduction. The difference between MDA-MB-231 cells and their supernatant was highly significant for both lagtime (p-value=0.0091) and peak (p-value=0.0039). FCM analysis showed a 10-fold decrease in TF+ MVs concentration between MDA and Sup MDA samples (Table I, 1113 versus 90/µl), suggesting that the majority of MVs detected by FCM carrying PCA activity remain associated to the cell surface and co-pellet with the cells after centrifugation at 4,500 g. These data are consistent with the reduction of PCA between these samples.

Since FCM cannot detect vesicles smaller than 300 nm, and since TEM showed a majority of MVs smaller than 300 nm, the link between the MVs size and PCA was evaluated through filtration of the supernatant using membranes with various cut-off sizes ranging from 0.65 to 0.1 µm (Fig. 4b). As compared to the initial supernatant (Sup MDA), which displayed a 9-fold reduction in lagtime, the 0.65 µm- or 0.45 µm-filtered supernatant (Sup MDA Filt 0.65 µm or 0.45 µm respectively) displayed a slightly reduced PCA, shown by a 7-fold reduction in lagtime when compared to the control. The difference between samples before and after the 0.65 or 0.45 µm filtration was highly significant both for lagtime and for the peak parameter. These data are supported by FCM as filtration at 0.65 or 0.45 µm reduced by 2-fold the number of TF+ MVs as compared to non-filtered supernatant (40 or 38 respectively, as compared to 90/µl) (Table I). Finally, lagtime was increased 2.2- and 2.5-fold after filtration through 0.22 and 0.1 µm, respectively, in comparison to unfiltered supernatant, but was still 4- and 3.6-fold reduced as compared to PBS. These analyses were performed without “MP reagent”; however, the same observations were made with or without addition of phospholipids. We conclude that TF-MVs of different sizes participate in PCA activity, those larger than 0.65 µm displaying proportionally less activity than the MVs retained by 0.22 and 0.1 µm filters, and the majority of the PCA was associated with components smaller than 0.1 µm.

The use of HTF-1, an anti-TF antibody, was associated with a strong inhibition of the PCA associated to MVs as revealed by a 6-fold increase in lagtime (Fig. 4c). On the other hand, the PCA was not affected by a non-blocking antibody (TF9-10H10), nor by an isotypic control (IgG1). These data confirm the specificity of PCA inhibition by the anti-TF antibody.

Inclusion of annexin V during the assay was associated with a complete abolishment of PCA (Fig. 4d).

The majority of PCA contained in supernatant was associated with the pellet after 100,000 g ultracentrifugation, but some activity remained in the post-ultracentrifugation supernatant (Fig. 4e): this supernatant still reduced lagtime 2.5-fold in comparison to NPP spiked with PBS, and however, it showed no significant increase of peak of active thrombin (Fig. 4e).

The lagtime and peak intra-assay variation coefficients were 12 and 11%, respectively. The lagtime and peak inter-assay variation coefficients were 14 and 50%, respectively.

### Influence of stirring and centrifugation on PCA of MDA-MB-231 cells and MVs

**Fig. 5:**
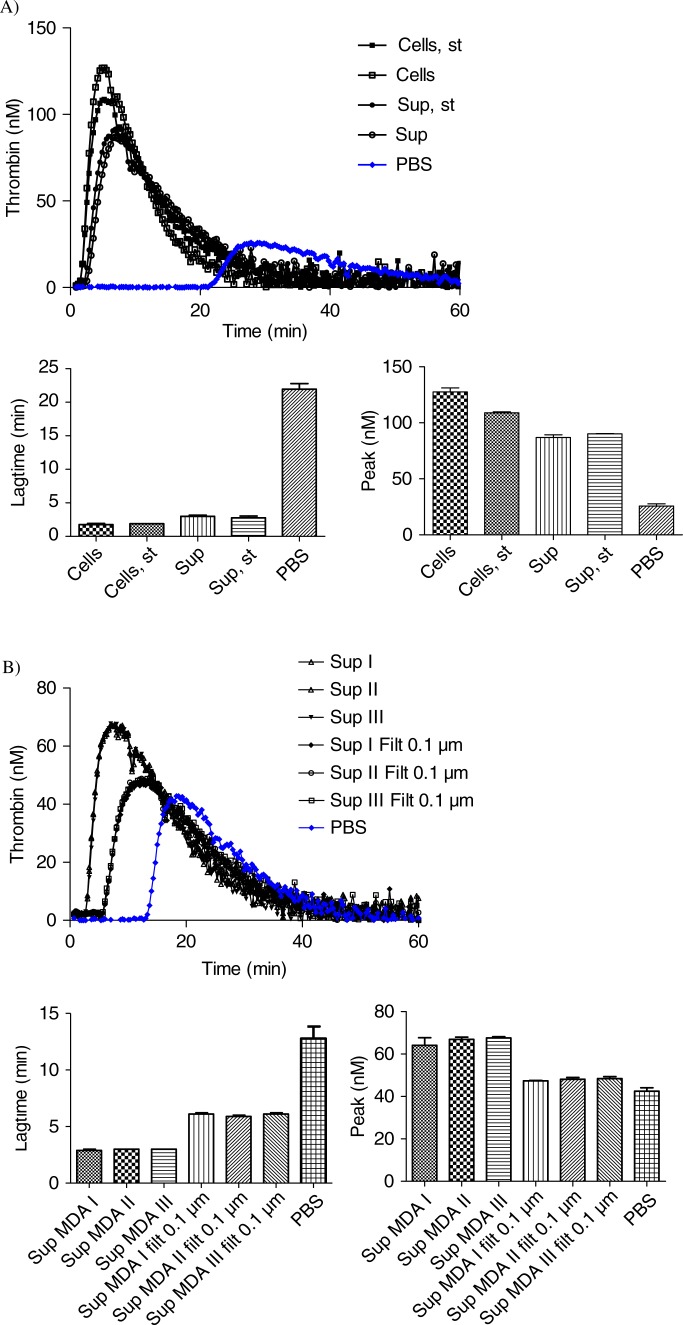
Effect of stirring during cell incubation and of cell centrifugation protocol on thrombin generation. A) Thrombin generation curves and histograms of lagtime and peak parameters of normal pool plasma (PBS) spiked with MDA-MB-231 cells stirred (Cells, st) or not (Cells) or with their respective supernatant (Sup, st and Sup). No difference in PCA is observed between the stirred and non-stirred samples; B) Thrombin generation curves and histograms of lagtime and peak parameters of normal pool plasma spiked with supernatant of cells filtered or not (Sup vs. Sup Filt 0.1 µm) and produced by 3 different centrifugation protocols (Sup I, II, III). In control wells, NPP is spiked with PBS. The curve is representative of 3 independent experiments.

The stirring of MDA-MB-231 cells during MVs generation does not modify the PCA of cells (Cells) and MVs (Sup) derived from 600,000 cells/ml (Fig. 5a). However, FCM analysis showed a difference between MVs concentration in samples whether stirred or not (Table I).

The centrifugation protocol used to separate cells and MVs does not significantly impact TGA curves (Fig. 5b). Indeed, curves are very similar both for the supernatant and the 0.1 µm-filtered supernatant generated from 600,000 MDA-MB-231 cells/ml, using either one of protocols I, II or III described in Fig. 1.

### Sensitivity of TGA

**Fig. 6:**
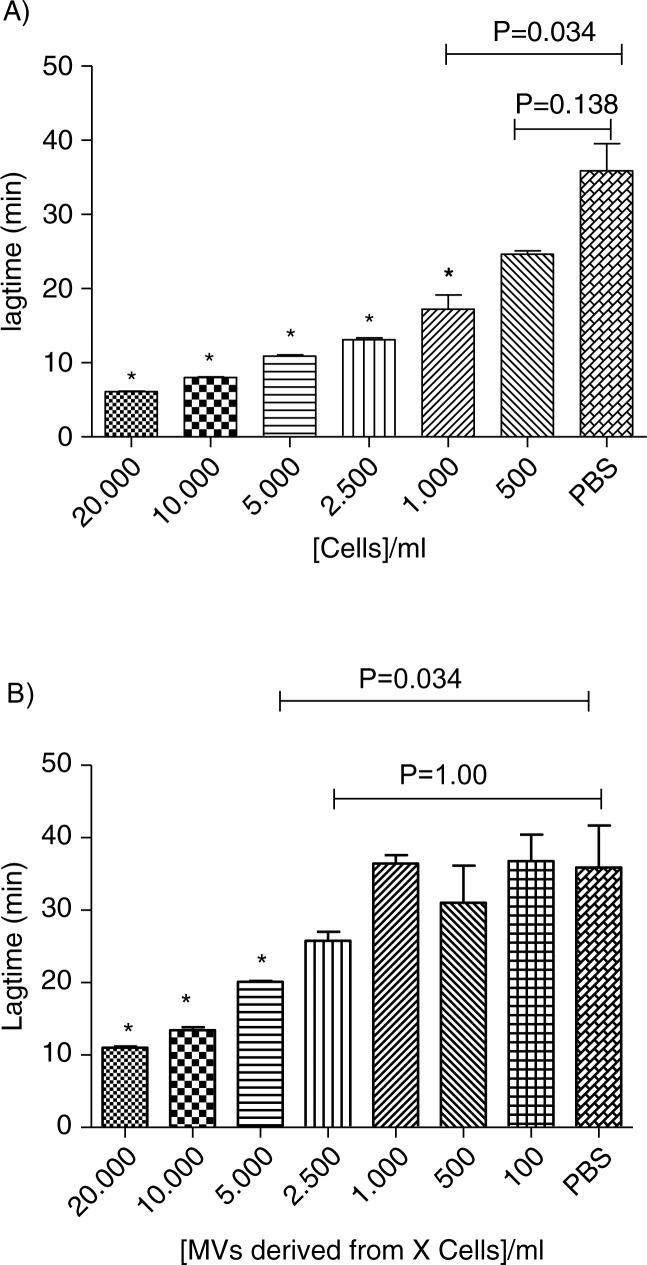
Procoagulant effect of cells (Cells, A) and MVs (Sup, B) derived from different MDA-MB-231 cell concentrations (0; 100; 500; 1,000; 2,500; 5,000; 10,000; and 20,000 cells/ml) on lagtime. In control wells, NPP is spiked with PBS. Results are presented as mean±SD. Data with significant difference in comparison to control are indicated with *(p<0.05).

As shown in Fig. 6, a dose-response relationship is observed from 100 to 20,000 cells/ml for both MDA-MB-231 cells and MVs. A threshold concentration of 500 MDA-MB-231 cells/ml is sufficient to release enough TF-MVs that significantly reduce the lagtime in comparison to control (p-value=0.034). When the supernatant is considered, 5,000 MDA-MB-231 cells/ml is the threshold concentration required to significantly reduce the lagtime (p-value=0.034). The coefficients of variation for Lagtime, ETP, Peak and Ttpeak were 12, 11, 11 and 9% in intra-assays (n=6) and 14, 34, 50 and 16% in inter-assay (n=6), respectively.

## Discussion

VTE prophylaxis during systemic chemotherapy is not recommended for ambulatory patients with cancer (23). Indeed, there is currently no validated biomarker presenting a thrombotic profile able to lead to an adapted prophylaxis (4). The circulation of active TF associated with MVs has been considered as a promising and interesting biomarker to prevent VTE in cancer (2, 5, 10). An ongoing phase III study (MicroTEC, NCT00908960) in advanced cancer patients with high levels of TF-bearing MVs has been designed to evaluate the benefit of primary thromboprophylaxis and to validate the prognostic usefulness of measuring TF-bearing MVs (6). However, validation and standardisation of techniques measuring TF-MVs are required before considering cut-off values of this biomarker in risk-stratification approaches. In this study, we compared results obtained with TEM, FCM and TGA to determine the size, the concentration and the PCA of tumour MVs. We used the well-established invasive/metastatic MDA-MB-231 breast carcinoma cell line, which is known to express high levels of TF (11). By FCM, we highlighted that the majority of cells and 10% of cell-derived-MVs are positive for TF, and only a few microvesicles express MUC-1 (Fig. 3 and Fig. S1). MUC-1 was chosen since it has been shown that TF-MV activity of patients with breast cancer who presented with acute VTE, correlated with the presence in the blood of MVs expressing the epithelial antigen MUC1 (17). Moreover, FCM allows determining TF+ MVs or MUC-1+ MVs concentrations. However, FCM suffers from a lack of sensitivity to small-sized MVs (i.e. MVs size <0.5 µm) (8), and it has been recently shown that microvesicle detection by FCM is attributed to both large single vesicles and swarm detection of smaller vesicles (24, 25). Therefore, data presented in Table I can represent both large vesicles and aggregates of small microvesicles. Although new tools using size-calibrated beads and recent progress in instrumentation may improve detection of MVs populations of smaller sizes, FCM is the only validated technique available for MV quantification. However, unless recent improvements, FCM is only suitable for large microvesicles (8, 26). Thus, FCM will not allow the detection of smallest MVs and does not give any information about their TF-mediated PCA.

Using TEM, we showed that there is a budding of MVs on cell surface, these results are in agreement with previous study on MV formation and MV constitution can be directly linked to the cell surface membrane. However, for vesicles away from cells, we cannot determine whether they bud off the cell membrane or whether they were released by another means. Moreover, by TEM, we showed that size of MDA-MB-231-derived MVs ranges from 0.05 µm to 0.45 µm in diameter, the majority of MVs is comprised between 0.1 µm and 0.2 µm and 36% of MVs is comprised under 100 nm. These results are in agreement with those of Xiong (27). However, as slices are performed, these measures may be underestimated as the vesicles are not always cut in their equator. On the other hand, as observed with manufactured nanoparticles (28), MVs may associate with proteins (corona effect) during TEM preparation leading to an overestimation of the size. Cryoelectron microscopy may represent an interesting alternative to view every MV in natural condition (29).

We also validated TGA to characterise the PCA of MVs. In agreement with FCM data, we confirmed that the PCA of MDA-MB-231 cells is mainly due to MVs release. The higher PCA observed for MDA-MB-231 cells compared to their supernatant mostly resulted from a loss of the largest MVs or MVs aggregates by centrifugation, as shown by the differences in MVs concentration between cells and supernatant determined by FCM. However, our study confirmed that MDA-MB-231 cells could also support PCA (30). Surprisingly, we also showed that the 74% of TF activity is associated with particles smaller than 0.1 µm even if only 36% of MVs visualised by TEM are smaller than 0.1 µm. The filtration experiments also show that about 8% of activity is associated with vesicles larger than 0.65 µm, vesicles of sizes comprised between 100 and 650 nm display 20% of MVs PCA. Thus very small vesicles display a high PCA.

By using an anti-TF function-blocking antibody (HTF-1) and annexin V, which links phosphatidylserine, we confirmed that the PCA of MVs is related to the expression of active TF and negatively charged PL (31). Moreover, annexin V block phospholipids from MVs contained in the NPP. The anionic phospholipids are required for initiation and propagation of TF-dependent coagulation. It seems therefore likely that only TF bound in negative phospholipids membrane will support thrombin formation. This result confirms that the active form of TF mainly comes from MVs. It has been extensively reported that the presence of TF on the cell surface does not necessarily correlate with TF activity. Indeed, the majority of membrane-anchored TF molecules would be in a non-active encrypted conformation and “decryption” would thus be essential for the expression of TF function (32). The difference in peak between supernatant of MDA-MB-231 cells and MVs can be explained by the phospholipids provided by tumour cells (11, 17).

Because the clinical importance of MVs is increasingly being recognized, it is crucial to standardise methods to allow comparison of data across different studies. To validate the use of TGA for measuring the PCA of MVs, we assessed the impact of 2 pre-analytical variables, that is, stirring and centrifugation, and determined the sensitivity of this technique. Contrary to a previous report (11), the stirring of cells did not increase the production of MVs, as shown by MV counting by FCM and above all, by PCA measurement. In addition, no significant differences between the 3 centrifugation protocols were observed in TGA, which is compatible with the small size of MVs released by MDA-MB-231. Moreover, ultracentrifugation did not allow abolishing completely the PCA, which is in contradiction with Davila et al. (11). One hypothesis is that some small TF-MVs may not be pelleted with ultracentrifugation at 100,000 g and may require higher speed or longer time of centrifugation. Another explanation can come from the cells’ release of active soluble TF (33). Finally, 5,000 MDA-MB-231 cells/ml are sufficient to detect a lagtime reduction mediated by TF-MVs when compared to NPP alone. This technique is more sensitive without reagent but the variability increased.

In vivo the correlation between TF-MV and thrombosis may become more or less strong depending on the type of tumour/cancer present in a patient (6, 7, 10). Therefore, additional in vivo studies examining the relationship between tumour/cancer progression, thrombosis and TF-MV is critical.

In addition, atomic force microscopy, a high-sensitive technique able to image biological samples in aqueous fluids, was recently proposed for the detection and quantification of CD41-positive MVs (34). This technique should be developed and validated for TF-MVs and compared with FCM, TEM and TGA.

## Conclusions

This is the first study that uses FCM, TEM and TGA to characterise MVs. MDA-MB-231 releases MVs whose size was 121±54 nm by TEM. TGA is a useful technique to study the PCA of tumour-cell-derived MVs whatever their size. It should be combined by high-sensitive sizing and counting techniques such as AFM in order to estimate the concentration of specific MVs (i.e. TF-MVs). TGA should be validated and used to study TF-MV in plasma from healthy subjects and patients.
